# A Review on Laser-Induced Graphene-Based Electrocatalysts for the Oxygen Reduction Reaction in Electrochemical Energy Storage and Conversion

**DOI:** 10.3390/nano15141070

**Published:** 2025-07-10

**Authors:** Giulia Massaglia, Marzia Quaglio

**Affiliations:** 1 Department of Applied Science and Technology, DISAT, Politecnico di Torino, Corso Duca degli Abruzzi 24, 10129 Torino, Italy; 2 Center for Sustainable Future and Technology, CSFT@Polito, Istituto Italiano di Tecnologia, Via Livorno 60, 10100 Torino, Italy

**Keywords:** laser-induced graphene (LIG), metal-free catalysts, electrochemical energy devices, energy storage devices

## Abstract

The increasing demand for efficient and sustainable energy conversion technologies has driven extensive research into alternative electrocatalysts for the oxygen reduction reaction (ORR). Platinum-based catalysts, while highly efficient, suffer from high costs, scarcity, and long-term instability Laser-Induced Graphene (LIG) has recently attracted considerable interest as an effective metal-free electrocatalyst for oxygen reduction reaction (ORR), owing to its remarkable electrical conductivity, customizable surface functionalities, and multi-scale porous architecture. This review explores the synthesis strategies, physicochemical properties, and ORR catalytic performance of LIG. Additionally, this review offered a detailed overview regarding the effective pole of heteroatom doping (N, S, P, B) and functionalization techniques to enhance catalytic activity. Finally, we highlight the current challenges and future perspectives of LIG-based ORR catalysts for fuel cells and other electrochemical energy applications. Furthermore, laser-induced-graphene (LIG) has emerged as a highly attractive candidate for electrochemical energy conversion systems, due to its large specific surface area, tunable porosity, excellent electrical conductivity, and cost-effective fabrication process. This review discusses recent advancements in LIG synthesis, its structural and electrochemical properties, and its applications in supercapacitors, batteries, fuel cells, and electrocatalysis. Despite its advantages, challenges such as mechanical stability, electrochemical degradation, and large-scale production remain key areas for improvement. Additionally, this review explores future perspectives on optimizing LIG for next-generation energy storage and conversion technologies.

## 1. Introduction

Several efforts were made to develop energy storage and conversion systems able to overcome the main problems correlated with the utilization of fossil fuels, such as greenhouse effects and environmental problems [1].

In particular, many researchers focused their attention on electrochemical energy storage or conversion technologies, like supercapacitors [2,3,4,5,6], water splitting [7,8,9] metal–air batteries [10,11], hydrogen technologies [12] and fuel cells, which played a transformative role in the global energy transition by enabling the large-scale adoption of renewable energy sources, representing the core of the European Hydrogen Strategy and RePower EU Plan [13].

The key process in some electrochemical devices, such as fuel cells, metal–air batteries, and other energy conversion technologies is represented by direct-oxygen reduction reaction (direct-ORR). Commonly, the ideal ORR catalysts rely on platinum-group metals, which are expensive and suffer from degradation over time [10,11,12].

The main bottleneck, common to all major renewable energy sources, is the need to use catalysts, such as noble metal materials, including Pt, Pd, Ir, and Ru etc., having superior electrochemical properties, but at the same time characterized by high cost and scarcity in environment [10,11,12].

Given these factors, it is reasonable to assert that the global demand for achieving high-efficiency and environmentally friendly conversion technologies has driven extensive research to reduce the amount consumed by noble metals or replace them with other non-precious metals, leading thus into advanced carbon-based materials.

In addition, the transitions towards renewable energy sources and the widespread adoption of electric vehicles require high performance energy storage devices with enhanced efficiency, stability, and environmental sustainability.

To satisfy all these requirements, carbon-based materials, particularly graphene and its derivates [14], have gained significant attention thanks to their properties, such as good electrical conductivity, mechanical strength, and chemical stability, combined with high specific surface area [15,16]. All these properties justified the employment of graphene in different fields, like catalysis [17,18], transparent electrodes [19,20,21], and sensors [22,23].

The use of graphene-based electrodes in several electrochemical energy devices allowed achieving excellent electrochemical performance by significantly limiting the use of noble metals [24].

Chemical vapor deposition and chemical exfoliation are considered as the common method involved to synthetize graphene material [25]. As deeply investigated in the literature, the main constraint of these two methods is represented by the required typical thermal treatment under high temperature pressure, specific atmosphere [25].

These traditional methods, indeed, often involve high production costs, toxic reagents, and complex processing steps, that hinder their large-scale applicability.

In this scenario, it has made mandatory the development of advanced technologies suitable to synthetize graphene-like materials in a facile, fast, safe, effective, and efficient way. Among all possible methods, direct laser scribing writing (DLW) played a pivotal role in employing a promising technique, able to ensure the formation of laser-induced-graphene (LIG) obtained by direct scribing onto the polymeric or biomass derived substrates under ambient condition, at room temperature and in a few minutes. This process ensured a localized transformation of the starting material into LIG material. Indeed, laser-induced-graphene (LIG) has emerged as a scalable and environmentally friendly alternative for electrocatalytic applications.

Therefore, laser-induced-graphene (LIG) has gained significant attention as a scalable and economical approach for producing graphene-like materials. First introduced in 2014, LIG is synthesized by direct CO_2_ laser irradiation of polymeric substrates in an air atmosphere, guaranteeing localized graphitization and the synthesis of a porous, electrically conductive graphene network. This method enables precise patterning of graphene structures on flexible substrates, making it highly suitable for energy-related applications, including supercapacitors, lithium-ion batteries, and fuel cells [26,27,28,29].

**Figure 1:**
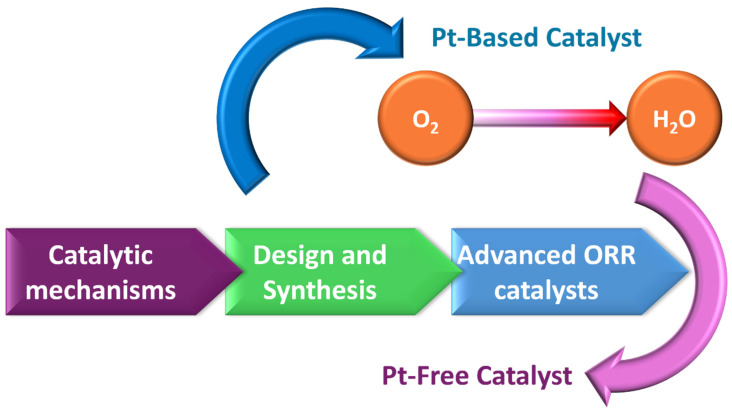
Representative scheme of Pt-based catalysts involved for ORR and, at the same time, the capability of finding new Pt-free catalyst to ensure the same electrocatalytic performance.

The attractiveness of LIG lies in its unique combination of properties. The high electrical conductivity of LIG facilitates efficient electron transport, essential for electrochemical reactions in energy storage and conversion devices. Additionally, its hierarchical porous structure provides a large surface area, enhancing ion and molecules diffusion. This property is essential to balance gas–water flows in devices as fuel cells, that, if not properly managed can negatively affect the overall performance of these energy conversion devices [29]. Additionally, doping LIG with heteroatoms like nitrogen (N), sulfur (S), phosphorus (P), and boron (B) can significantly enhance its ORR activity by altering the electronic structure and facilitating oxygen adsorption. All these combined properties make LIG materials an attractive electrocatalysts for ORR. All these strategies represent an innovative, efficient, and novel route to synthetize Pt-free catalysts, achieving the same electrocatalytic performance of Pt-based catalysts, as represented in Figure 1.

This review provides a comprehensive analysis of LIG as a metal-free electrocatalyst for ORR, focusing on its synthesis methods, physicochemical characteristics, catalytic mechanisms, and potential applications in fuel cells and renewable energy systems. Additionally, we discuss the challenges that must be addressed to enable the practical implementation of LIG-based ORR catalysts.

## 2. Synthesis and Properties of Laser-Induced-Graphene

### 2.1. Laser Processing Techniques for LIG Fabrication

**Figure 2:**
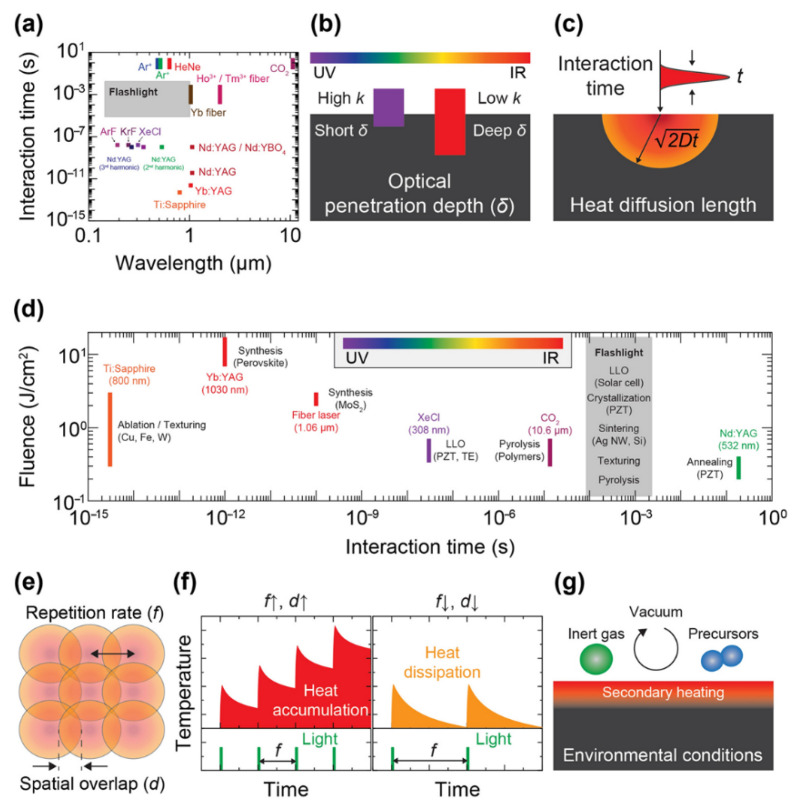
(**a**) The wavelength and interaction time characteristics of lasers and flash lamps [31,32,33,34,35,36,37,38,39,40,41,42,43,44,45,46,47,48,49,50,51,52,53]; (**b**) the effect of light wavelength on the optical penetration depth in light absorbing materials; (**c**) the role of interaction time in defining heat diffusion length; (**d**) time regime and its influence on LIG formation processes [45,46,47,48,49,50,51,52,53]; (**e**) the repetition rate and spatial overlap of laser pulses, which contribute to (**f**) heat accumulation or dissipation effects; (**g**) environmental conditions that initiate physicochemical reactions.

As deeply investigated in the literature, during these years, laser-induced-graphene (LIG) results to be an innovative emerging three-dimensional porous material obtained by laser irradiation of specific polymer substrates [30]. Direct laser writing (DLW) allows for microscale and nanoscale patterning, making it suitable for a wide range of applications, spanning from industrial use to life sciences. The main working principle was driven by photothermal and/or photochemical reactions triggered by a focused laser beam [31,32,33,34,35,36,37,38,39,40,41,42,43,44,45,46,47,48,49,50,51,52,53], which enables the conversion of a minute area into carbonaceous material to be achieved with such accuracy that it is difficult to achieve when implementing convention thermal treatments, as deeply reported in Figure 2. These localized reactions were strictly correlated with the laser process parameters and material properties.

Furthermore, as the mechanism of graphene synthesis is affected by both the properties of the laser and carbon precursors, wavelength and pulse duration are the critical parameters that significantly impact laser–material interactions. In most cases, these interactions resulted to be in a photo-thermal effect, since the target material absorbs the incident photon energy and transforms it into heat.

**Figure 3:**
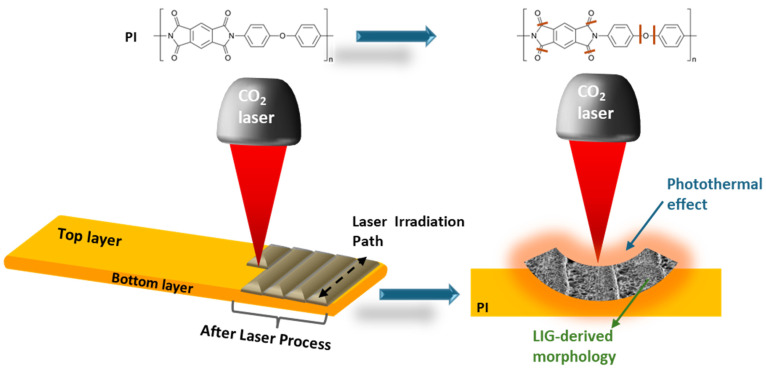
Scheme represented the photothermal effects induced by long-pulsed lasers characterized by long-wavelength (i.e., 10.6 µm, known as the CO_2_ laser).

This swift deposition of energy deposition brings about a significant local temperature rise, responsible for the carbonization, graphitization, and exfoliation process required to form LIG [54]. In this scenario, indeed, the breaking of bonds among atoms making up the polymer chain was mainly due to the heat transfer process, which also involves and induces lattice vibrations. While some atoms undergo recombination, others are released as gaseous by-products, primarily aromatic compounds (e.g., PI), which progressively evolve into nanostructures resembling graphene lamellae [55], as reported in Figure 3.

This effect typically occurred when a long-pulsed lasers, characterized by long-wavelength (i.e., 10.6 µm) was implemented, leading thus to conclude that the CO_2_ laser process is typically involved in transforming some polymeric precursor into LIG material [55].

**Figure 4:**
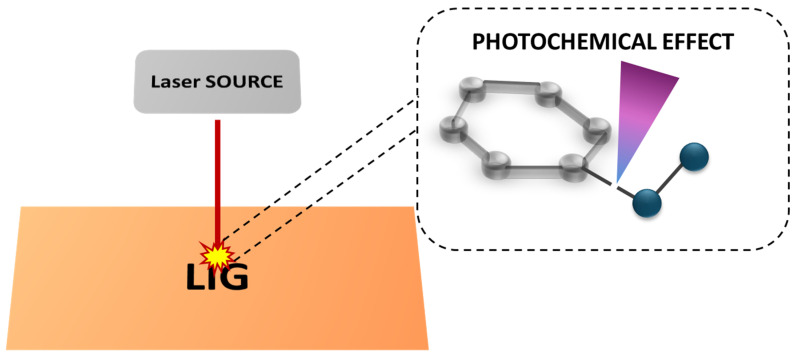
Representative scheme of photochemical effects induced on polymeric materials and responsible of the production of LIG materials.

On the contrary, with short-pulsed lasers and a short wavelength in the UV regions (i.e., 386 nm, 355 nm), the main effect induced by laser irradiation was a photochemical one. In this scenario, when the photon energy of UV lasers is comparable and/or greater than the chemical bond energy of the material, the adsorbed photon energy leads to the cleavage of C-H, C-O, C=O, and C-N bonds within the polymer, as described in Figure 4.

The main consequences of this chemical process were the release of gases, like CO, CO_2_, HCN, and C_2_H_2_, and the recombination of aromatic groups, leading thus to the formation of hierarchical porosity into LIG materials [56].

Ultrafast laser induction, occurring on sub-picosecond timescales, enables a non-thermal phase transition by generating an electron-hole plasma with enough density to overcome the sp^3^-C to sp^2^-C energy barrier. Unlike continuous-wave and long-pulsed lasers, which induces graphitization through a thermal phase transition, ultrashort laser pulses allowed for LIG formation without the requirement of high temperature annealing, strongly reducing the graphitization temperatures of the carbon source [57].

This rapid energy deposition minimizes heat dissipation into the surrounding environment, leading to localized extreme temperatures and stresses that drove the structural transformation into graphene [58].

It was possible to distinguish three different stages achieved when the femtosecond laser-induced-graphene process was applied [59]: (i) laser-assisted carbonization achieved at temperatures ranged from 250 °C to 500 °C, effective for the decomposition of carbon precursors into amorphous carbon; (ii) graphitization, occurring at 3000 K, reached when sp^3^-C is converted into sp^2^-C and the consequent aromatic network of graphene is formed; (iii) exfoliation induced when the temperature is higher than 20,000 K.

Finally, when the visible lasers (wavelength of 405 nm) were employed to ensure LIG formation, the combination of photothermal and photochemical effects occurred [60].

**Figure 5:**
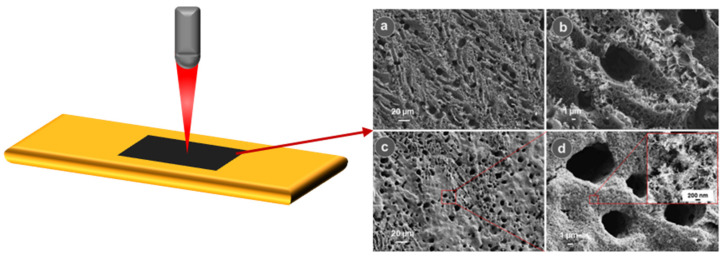
Morphological characterization typical of LIG materials, in which it is possible to evaluate the formation of a highly intricate fiber-like or lamellar porous structure within LIG. (**a**) SEM Images highlighting the lamellar porosus structure of LIG; (**b**) Morphological properties of LIG porosity; (**c**) deep investigation of pores’ structure (**d**) SEM images of a typical intricate fiber-like structure within LIG, magnification into the red box. Ref. [62] Reprinted (adapted) with permission from {Gosh. A. Kaur S., Verma G., Dolle C., Azmi R., Heissler S., Eggeler Y.M., Mondal K., Mager D., Gupta A., Korvink J.G., Wang D.Y., Sharma A., Islam M. ACS Appl. Mater. Interfaces 2024, 16, 40313−40325}. Copyright {2024} American Chemical Society.

During laser processing, the polymer surface experienced intense localized thermal shock, coupled with thermal expansion aiding gas release, which led to the development of a highly intricate fiber-like or lamellar porous structure within LIG [61], as shown in Figure 5.

The main parameters of laser processes, like power, focal focus, speed, and space of scanning, played a pivotal role in tuning crystal size and defect density of LIG. Owing to the strong short time of the sp^3^-C to sp^2^-C conversion, the carbon rings have limited time to reorganize into a perfectly ordered 2D lattice structure.

In conclusion, several works in the literature demonstrated how CO_2_ lasers, characterized by a wavelength of 10.6 µm, resulted in being more effective transforming some polymeric materials into LIG samples [63].

### 2.2. Carbon Sources for LIG-Based Electrodes

The design of LIG from several commercial polymers were evaluated during the last decades [64], leading to an overview on the gas evolution, surface area, electrical conductivity, and yield of LIG.

LIG is graphene-like material obtained by laser ablation applied on PI sheets (Kapton) [62,65].

In 2018, Ye et al. demonstrated the pivotal role of commercial CO_2_ laser writing as a simple and scalable method for fabricating and shaping three-dimensional porous graphene on PI films in an air atmosphere [66].

These results allowed the confirmation of the possibility to employ LIG sample in a fast and effective way, providing competitive advantages with respect to the traditional methods implemented to synthetize 3D graphene.

Furthermore, it was important to underline that only a certain class of polymers can be directly converted into LIG by laser process that occurred under air conditions. These polymers belong to the high-temperature engineering plastics, such as thermoplastics polymers [66], phenolic resins [67], biopolymers like lignin [68] and even textiles, wood, and food, for which in some cases, an inert environment is needed [69].

On the contrary vinyl polymers and polymers with lower melting point undergo a depolymerization and ablation rather than being converted into LIG when the CO_2_ laser process was applied under ambient conditions. For those polymers, an inert atmosphere was required to ensure the correct conversion into graphene-like materials. These requirements were completely avoided thanks to a new and innovative process [70], which leads these synthetic polymers to the conversion into graphene-like materials under an ambient atmosphere.

**Figure 6:**
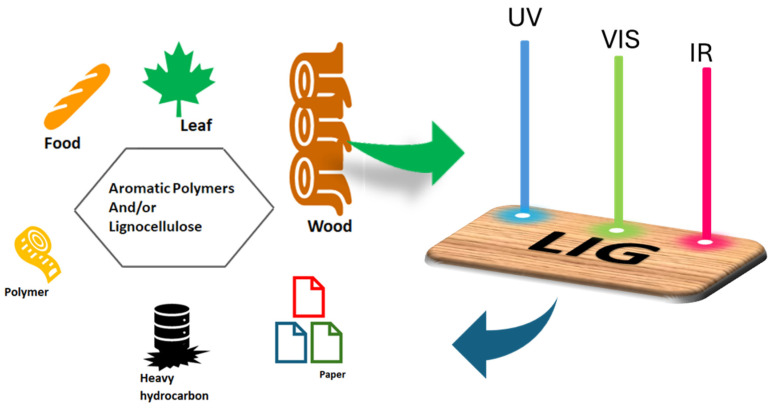
Photothermal reactions involved during the CO_2_ laser process that lead to the transformation of aromatic polymers and lignocellulose materials into LIG samples.

Several works in the literature demonstrated how the irradiation of CO_2_ lasers and long-pulsed lasers were effective in employing 3D porous graphene architectures from high-carbon content materials, including aromatic polymers [50,71] and lignocellulosic materials [69,72,73], through laser-induced photothermal transformation, as reported in Figure 6.

In addition to polyimides (PI, Kapton), several works in the literature confirmed the effectiveness of the CO_2_ laser process to transform some aromatic polymers into LIG, including polyether-ether-ketone (PEEK) [74] poly(ether imide) (PEI) [59,68], different polysulfones (PSU) [75,76], and lignin [77].

In this scenario, carbon precursors absorbed the laser energy irradiated on them, inducing a consequent lattice vibration. Moreover, the cleavage of covalent bonds within the precursor were induced by intense local heating, leading to gas release and the re-arrangement of residual carbon. As a result, porous 3D LIG structures were formed.

#### 2.2.1. Aromatic Polymers Transformed into LIG Materials

The capability to transform aromatic polymers, such as PI-films, into porous LIG materials, were deeply investigated by Tour et al. [66], which demonstrated the capability to employ a final porous graphene structure after exposing this polymeric film to an infrared (IR) CO_2_ laser under ambient conditions. It was possible to appreciate a morphological property of obtained LIG similar to a porous foam structure, due to the rapid generation of gaseous products during the laser process. The employment of the CO_2_ laser, thanks to its long wavelength (10.6 μm) and large pulse duration (14 μs), can induce the deposition of a consistent content of photothermal energy inside the materials. Since the heat does not have sufficient time to dissipate, this results in a localized reaction characterized by high pressure and temperature. These extreme conditions break C=O, C–O, and N–C bonds in PI, leading to the release of certain molecules as gases, while the remaining aromatic compounds undergo rearrangement to form graphene-like structures. The generated high-pressure gases were instrumental in enabling the efficient conversion of PI films to LIG in an air atmosphere:

High pressure suppresses the thermal degradation of carbon sources, allowing the formation of graphene aggregates from merging with the small carbon clusters.

All gas by-products released avoided, as much as possible, the oxidation of the graphitic structures during this laser process.

As deeply investigated by Lin et al. [78], other important effects on LIG formation from PI were directly correlated to pressure and environmental conditions. At high temperature and pressure, equal, respectively, to 2400 K and 3 GPa, it was possible to eliminate all non-carbon compounds as volatile gases, while carbon clusters remained in solid form. On the contrary, at a reduced pressure of 27 MPa, no graphene aggregates were detected and the PI decomposed into molecules and gases, such as CO and HCN.

The laser-induced graphitization of polymers was strongly affected by the fundamental structural units in the polymer chains. Polymers rich in aromatic (or imide) repeating units, such as polyimide (PI) and polyetherimide (PEI), facilitate the conversion into LIG. Conversely, non-aromatic hydrocarbons undergo rapid depolymerization and complete degradation at high temperatures without forming graphitized structures.

Green et al. [79] evaluated the behavior of all polymers characterized by a great potential to produce LIG, such as PI, PEI, polybenzimidazole (PBM), polycarbonate (PC), and poly-ether ether-ketone (PEEK). Their findings revealed the capability of laser irradiation process to induce the formation of amorphous structures during the initial phase of process (0.2 ns), and the consequent evolution into graphene-like structures during the whole process. Additionally, during the transformation of polymers into amorphous carbon, molecular CO gas is rapidly released, while H_2_ evolution takes place as LIG transitions into an enhanced crystalline structure.

Furthermore, polyetherimide and poly-ether ether-ketone showed an high yield LIG, characterized by large surface area and porosity, whereas polycarbonate employs LIG showing five- and seven-membered rings.

#### 2.2.2. Lignocellulose as Initial Substrates to Obtain LIG

In 2017, Tour et al. [80] demonstrated that exposing pinewood to a 10.6-μm CO_2_ laser can convert it into porous graphene. The transformation of wood into LIG materials, using a CO_2_ laser, was influenced by three main factors: (1) the presence of an inert atmosphere, (2) the lignin content and its structure, and (3) the laser power.

Initially, the CO_2_ laser was implemented under a mixture of an argon and hydrogene (Ar/H_2_) atmosphere because direct laser irradiation in the air caused wood ablation instead of LIG formation. This ablation occurs due to oxygen-induced combustion, which causes the complete decomposition of wood components, including cellulose, hemicellulose, and lignocellulose.

Secondly, cross-linked lignocellulose with a high lignin content is critical for producing LIG. Woods rich in lignin, such as pinewood, are more suitable for generating high-quality LIG compared to those with lower lignin levels, since lignin contains abundant aromatic subunits that readily convert into graphene during thermal treatment. The structural arrangement of lignin composites in wood also plays a pivotal role in LIG formation. For example, commercial lignin, which lacks a cross-linked structure and tends to from a viscous, oil-like substance, rather than LIG, when exposed to laser irradiation. Wood is composed of dense one-dimensional crystalline cellulose embedded within a cross-linked lignin and hemicellulose matrix, making this unique structure essential for successful LIG synthesis.

Third, although increasing laser power improved LIG quality, excessive laser power (above 90%) overheated the wood, leading to a larger number of amorphous structures into LIG.

Beyond wood, various other materials with lignin content are also suitable for conversion into LIG. For example, coconut shells, cork, and potato skins, containing approximately 25%, 30%, and 36% lignin, respectively, were transformed into LIG in the air thanks to CO_2_ laser repeated irradiations characterized by a low power, which was close to 5%. On the contrary, it is important to underline how the same irradiation conditions can be detrimental for wood, which tends to burn or become ablated [14]. All these results make it possible to state that atmospheric control is no longer a fundamental parameter for the formation of LIG material, greatly simplifying the process of producing graphene-like material from samples containing lignocellulose

### 2.3. Heteroatom Doping LIG

In addition to investigating the effects of laser parameters, controlled atmospheres, and various laser-induced graphitization processes of LIG, increasing research efforts have been directed toward heteroatom and nanoparticle (NP) doping. Specifically, LIG doping plays a crucial role in enhancing device performance by introducing additional atomic defects and functional groups. To this purpose, indeed, the presence of some heteroatoms proved to be an effective method for modifying the electron distribution of both the dopant and neighboring atoms, leveraging the differences in size and electronegativity among carbon and the heteroatoms [81].

Moreover, the presence of heteroatoms within carbon lattices affected also the physicochemical properties of final graphene-like materials.

Among the reported methods, doping strategies can be broadly categorized into two main approaches. A widely adopted technique is the one-step in situ laser modification, where poly-(amic-acid) (PAA) is blended with a doping precursor prior to laser irradiation. Alternatively, the two-step modification method involves first generating LIG materials on PI film, followed by post-treatment with doping precursors.

Among all possible heteroatom-doped LIG, the most common reported were nitrogen-doped LIG (N-LIG), phosphorous-doped LIG (P-LIG), fluorine-doped LIG (F-LIG) and sulfur-doped LIG (S-LIG), resulting in an effective strategy to tailor their electrochemical properties, enhance their electrical conductivity and surface wettability. In this scenario, moreover, many works in the literature demonstrate the importance of presence of those heteroatoms to enhance the electrocatalytic activity of LIG towards direct oxygen reduction reaction (ORR).

#### 2.3.1. N-Doped LIG

It is widely known in the literature of the importance of nitrogen doping to improve the electrocatalytic properties of a carbonaceous material, applied, for example, as a Pt-free catalyst for direct oxygen reduction reaction (ORR).

In this context, the development of winning strategies to combine the fast, effectiveness and repeatability of the laser process to obtain/employ LIG materials, with the ability to form N-doped LIG, could be of great interest in the scientific community.

**Figure 7:**
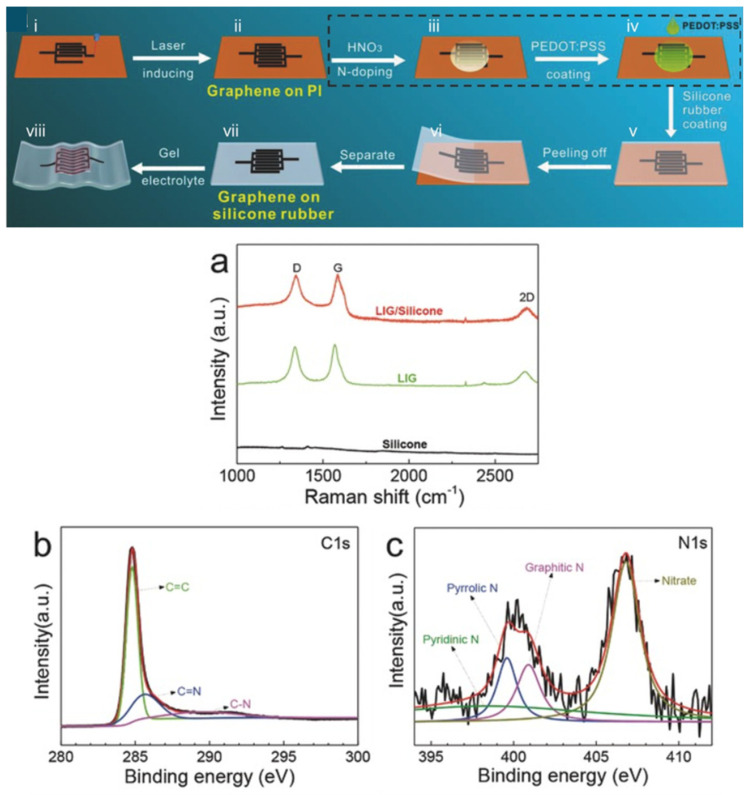
(**i**–**viii**) Scheme of preparation of patterned LIG using as active material on silicone rubber substrates: (**i**,**ii**) design of patterned LIG onto PI sheets; (**iii**) the subsequent immersion in HNO_3_ to induce the formation of nitrogen doping inside LIG materials; (**iv**) coating of LIG substrate with PEDOT:PSS to improve not only the electrical conductivity but also the capacitance of the final devices; (**v**–**viii**) represents all experimental phases implemented to transfer as designed LIG electrode to silicone rubber, ensuring the proper mechanical flexibility. The graphs from (**a**–**c**) represented the physic-chemical characterizations implemented to demonstrate the possibility to design a N-doped LIG materials with this approach: (**a**) Raman spectroscopy identified LIG material with structural defects; (**b**,**c**) C1s and N1s spectra survey suitable to identify nitrogen doping achieved. Ref. [83] Modified figure reprinted with the permission from {Weixing Song, Jianxiong Zhu, Baoheng Gan, et al. Flexible, Stretchable, and Transparent Planar Microsupercapacitors Based on 3D Porous Laser-Induced Graphene. Small 2017, 14}. Copyright {2017} John Wiley and Sons.

Song et al. [82] demonstrated the capability to tune N-doped LIG by immersing LIG samples, obtained by implementing direct CO_2_ laser writing on PI sheets, in a solution of 10 M HNO_3_ for 2 min. Figure 7 reports showing physic-chemical characterizations that demonstrated that obtained samples were graphene-like materials doped with nitrogen heteroatoms. All achieved results confirm the effectiveness of this wet chemical method, involved in ensuring nitrogen doping LIG. Indeed, it was possible to verify through Raman spectroscopy that the obtained sample was composed of LIG flakes with structural defects and numerous graphene edges, suitable to improve the accessible surface area and therefore better electrochemical performance. Furthermore, XPS spectroscopy confirmed the achievement of N-doping within LIG materials, by identifying the presence of main peaks, attributed to pyridinic-N (398.3 eV), pyrrolic-N (399.6 eV), graphitic-N (401.2 eV), and nitrate (406.8 eV).

Kim et al. [84] investigated a densification of LIG (d-LIG), which showed nitrogen doping, achieving a promising approach to deeply enhancing the LIG performance across the electrocatalysis application area.

**Figure 8:**
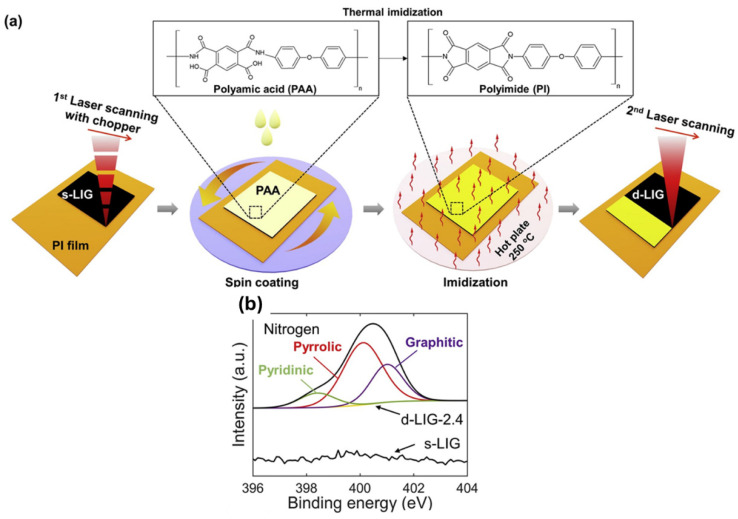
(**a**) Schematic representation of experiments involved to achieve a densification of LIG, characterized by N-doping; (**b**) N1s survey spectra underlined the presence of peaks related to Pyridinic-N (398.2–398.4 eV), pyrrolic-N (400.0–400.1 eV), and graphitic-N (401.0–401.7 eV). Ref. [85] Modified figure reprinted from {Simultaneous densification and nitrogen doping of Laser-induced-graphene by duplicated pyrolysis for supercapacitor applications, 441, Kim et al.}. Copyright {2019} with permission from Elsevier.

This approach (Figure 8a) involves an initial photothermal pyrolysis achieved by laser beam interaction with the polyimide sheet. Subsequently, the thus-obtained LIG sample was soaked in a poly (pyromellitic dianhydride-co-4,4-oxydianiline) amic-acid solution (PAA, 12.8 wt%, in N-methylpyrrolidone) for 10 min under ambient conditions. This process leads to the formation of a PAA film on the LIG after the removal of the excess PAA. To eliminate any residual solvents, an annealing treatment must be implemented at 100 °C for 1 h in a vacuum oven. To complete the imidization, the sample underwent additional annealing treatment at 250 °C for 30 min. At this stage, a second laser pyrolysis was implemented.

With the main aim to demonstrate the self-induced nitrogen-doping inside d-LIG, XPS characterizations were implemented, confirming the identification of three peaks, mainly correlated with the presence of pyridinic-N (398.2–398.4 eV), pyrrolic-N (400.0–400.1 eV), and graphitic-N (401.0–401.7 eV).

Wan et al. [86] proposed a self-induced N-doped LIG, starting from PI sheets, thanks to a high/large amount of nitrogen inside the polymeric chain before laser process. They demonstrated that the capability to achieve N-doping was close to 2.4% and 4.5% in the graphene skeleton in the forms of pyrrolic nitrogen and graphitic one.

**Figure 9:**
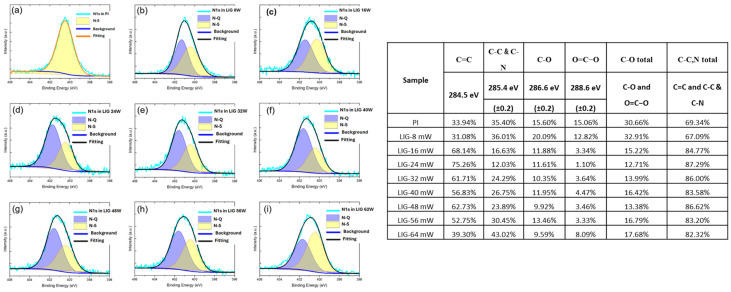
XPS analysis of N1s implemented on different LIG materials, with the aim at correlating the CO_2_ laser process parameters with the variation of nitrogen amount inside final LIG materials, correlated with the different laser power: (**a**) and LIG 8 W (**b**), 16 W (**c**), 24 W (**d**), 32 W (**e**), 40 W (**f**), 48 W (**g**), 56 W (**h**), 64 W (**i**) [86].

They confirmed an active role of laser parameters that are able not only to tune the amount of N-doping inside LIG materials, but also to modulate the relative proportion between graphitic and pyrrolic nitrogen, leading to deeply affect the electrical and electrochemical properties of the LIG, as reported in Figure 9.

#### 2.3.2. Oxidized LIG (LIG-O)

**Figure 10:**
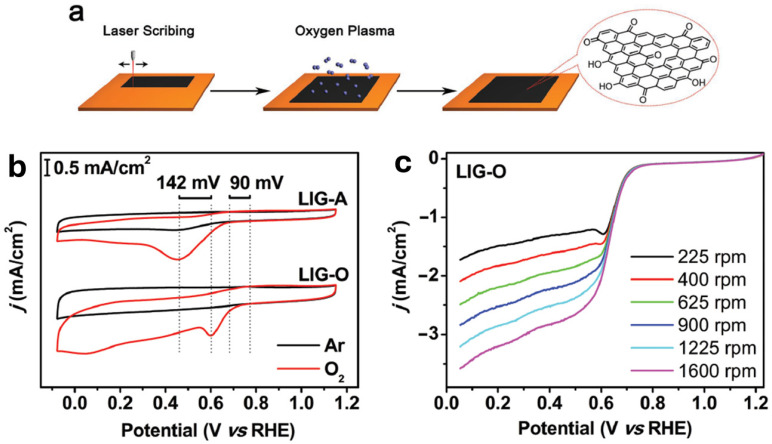
(**a**) Scheme for the preparation of LIG-O; (**b**) CV curves of LIG-O recorded in 0.1 m KOH with Ar or O_2_ bubbling at 50 mV s^−1^, compared with the LIG without plasma treated (LIG-A); (**c**) LSV curves of LIG-O at different rotating speed in 0.1 m KOH with O_2_ flux [87].

Zhang et al. [87] introduced an effective metal-free catalyst for both oxygen evolution and reduction reactions, employing oxidized laser-induced-graphene (LIG-O), as depicted in Figure 10a.

The oxidation of LIG through O_2_ plasma treatment enhances its performance in the oxygen evolution reaction (OER), allowing the reduction of onset potential of 260 mV and a Tafel slope of just 49 mV dec^−1^, while also improving activity in the oxygen reduction reaction. The presence of oxygen-containing functional groups plays a crucial role in creating active sites, and, at the same time, promoting the adsorption of OER intermediates and reducing activation energy.

In this work, the oxidized LIG-O was applied as a highly effective catalyst for oxygen electrocatalysis. The oxidation of LIG, which inherently possesses a large surface area, generates numerous active sites that significantly enhance electrocatalytic performance, resulting in excellent OER and ORR activity (see Figure 10b,c). Furthermore, the exceptional OER activity of LIG-O is attributed to the presence of functional groups characterized by the presence of oxygen (e.g., C=O), which improve the adsorption of OER intermediates and accelerate the rate-limiting step. With its combination of high efficiency, low cost, and simple fabrication, LIG-O presents itself as an optimal substitute for metal-based catalysts in applications including water splitting, metal–air/O_2_ batteries, and beyond. These offers important insights into the catalytic features of LIG-derived materials and supporting ongoing improvements in the catalytic performance of surface-oxidized carbon nanomaterials.

#### 2.3.3. Co-Doped LIG

It was widely accepted by the research community that co-doped graphene or graphene doped with two different heteroatoms played a pivotal role in improving electrocatalytic properties of final synthetized materials. Among all possible combinations of heteroatoms doping, the involvement of N/B, N/S and N/P heteroatoms as co-dopants were suitable to increase the electrochemically active sites of graphene.

Khandelwal et al. [83] implemented a flexible and cost-efficient approach for the simultaneous co-doping of LIG with nitrogen (N) and phosphorus (P) heteroatoms through two steps of laser irradiation. In particular, the second laser irradiation was implemented onto the modified surface of LIG materials by forming a thin film of PAA mixed with metaphosphoric acid (HPO_3_), which it was subsequently subjected to an imidization process through an annealing treatment.

**Figure 11:**
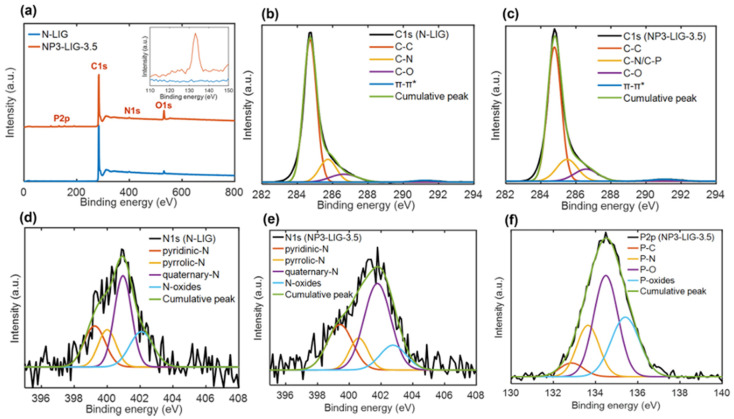
XPS spectra of N-LIG and NP3-LIG-3.5. (**a**) Survey and expanded survey scan spectra in the binding range of 110–150 eV (inset). (**b**,**c**) High-resolution C 1s. (**d**,**e**) N 1s spectra of N-LIG and NP3-LIG-3.5, respectively. (**f**) High-resolution P 2p spectrum of NP3-LIG-3.5 (**f**) [83].

The amount of the P-dopant precursor and the laser power applied during the second pyrolysis stage played a critical role in producing good-quality LIG with a correct balance of nitrogen and phosphorus atoms inside the LIG and enough electrochemically active sites, as evidenced by XPS spectra reported in Figure 11. The synergistic interaction between N and P heteroatoms significantly enhanced electrochemical performance, surpassing that of both mono-doped and undoped counterparts.

The same effective strategy was implemented to achieve a facile and versatile approach for the synthesis of nitrogen (N) and boron (B) co-doped LIG (NB-dLIG) [83].

After the initial laser pyrolysis, the LIG is covered by thin layer of polyamic-acid (PAA) and H_3_BO_3_, followed by an additional laser irradiation. This process leads to both structural densification and the simultaneous presence of N and B heteroatoms into the graphene-like LIG framework, characterized by an interconnected porous structure. The amount of H_3_BO_3_ and laser parameters for the second laser pyrolysis played a crucial role in tuning the amount of N and B heteroatoms inside LIG, achieving enhanced performance if compared with the N-doped LIG and/or undoped-LIG.

Differently from all previous works that focused their attention on the formation of doped and/or Cod-doped LIG, starting from PI sheets. Sing et al. [75] proposed the generation of sulfur-doped LIG by implementing laser irradiation of PSU-polymer class in a only one step and through a solvent and reagent free technology. Yang et al. [85] enabled rapid doping of sulfur atoms and MoOx nanoparticles modification on LIG surface. To achieve this final goal, CO_2_ laser writing was implemented on a film made of sulfonated poly(ether ether ketone) (SPEEK) mixed with molybdenum pentachloride (MoCl_5_). The presence of sulfur doping fulfilled two important functions: (i) enhancement of defect state density of graphene, facilitating electron transport and (ii) the positive contribution to the photothermal effect of MoOx.

#### 2.3.4. In Situ Formation of Metal Oxide Nanoparticles

It is well known in the literature how the efficiency of LIG material toward ORR is strictly correlated with the half reduction of oxygen, which required two electron pathways, producing O_2_ and OH^−^ products characterized by a half potential (E1/2) close to 0.66 V.

To overcome all these limitations of pure LIG and with the main aim to ensure direct oxygen reduction reactions substituting or limiting the use of metal noble catalysts, several researchers focused their attention on the formation in situ of metal oxide nanoparticles embedded in LIG [88]. Ye et al. demonstrated the capability to design metal oxide nanoparticles embedded in LIG materials, with a metal loading lower than 1%. The presence of metal oxide crystals positively affects the electrocatalytic properties of LIG towards ORR, leading to tuning these properties by changing the metal complex precursor or doping with other elements. To ensure the presence of metal oxide (MO) nanoparticles inside the LIG materials, a CO_2_ laser writing was implemented on a metal complex (MC) PI sheet, of which the surface was properly modified.

**Figure 12:**
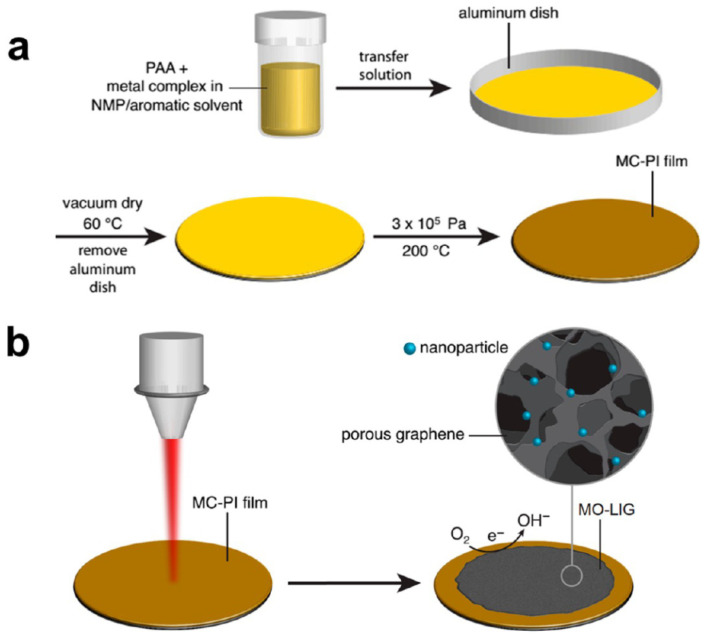
Schematic representation of experiments involved to achieve the formation of metal oxide nanoparticles embedded in LI. (**a**) reported the experimental scheme followed to obtain a final MC-PI film; (**b**) CO_2_ laser scribing implemented to synthetize the final MO-LIG samples, confirming the capability of laser writing to ensure the simultaneous transformation of polymeric film into LIG samples and the nanoparticles of MO onto the surface of LIG [86].

As reported in Figure 12, MC-PI was obtained by metal-complex-containing PAA solution. A MC-PAA film forms through solvent evaporation from metal-complex-containing PAA solution placed in an aluminum dish. Subsequent dehydration of PAA by heating at 200 °C under pressure results in the formation of MC-PI.

## 3. Application of LIG-Based Electrocatalysts for Renewable Energy Sources

### 3.1. Renewable Energy Storage and Conversion Systems

One of the central issues in today’s world is how energy is produced and used efficiently. Energy storage systems have become essential tools for capturing generated energy and making it available when needed. Many works in the literature underlined the importance of integration between energy conversion and energy storage systems [89,90]. The capability to develop new technology able to use “green” energies results to be essential to satisfy all energy needs and environmental sustainability. Nevertheless, a significant limitation of these “green” energy sources is their reliance on the availability of natural resources, such as sunlight, wind, or geothermal heat, which often does not coincide with actual energy demand. To overcome these limitations, the integration of energy storage and energy conversion systems proves to be vital, suitable to support modern infrastructure and enable the shift toward renewable energy sources. Among all electrochemical energy storage devices, the metal–air (M–Air) batteries have attracted significant attention as a potential energy storage system [89]. These devices offer exceptionally high energy density compared to conventional battery technologies, making them well-suited for long-duration energy storage and applications where lightweight energy sources are essential.

Moreover, many M–Air Batteries (i.e., zinc–air, aluminum–air) utilize metals that are inexpensive, abundant, and environmentally friendly, contributing to both scalability and sustainability [89].

A further advantage arises from their use of atmospheric oxygen, dissolved in air, as cathodic reactant, which eliminates the need to store both electrodes internally and significantly reduces battery weight. On the other hand, regarding the necessity to develop energy renewable technologies, being able to transform/transduce different types of energies embedded in a fuel directly into electrical energy, fuel cells (FCs) are gaining increasing attention as a renewable energy solution, due to their high efficiency, low environmental impact, and versatility across a wide range of applications. Unlike conventional combustion-based systems, fuel cells can convert chemical energy directly into electrical energy with minimal losses, resulting in greater energy efficiency [91].

Moreover, when hydrogen produced from renewable sources is used as fuel, the only byproduct is water, making the process entirely clean and sustainable.

Another key advantage is their scalability and modularity; fuel cells can be employed in small-scale portable devices as well as in large-scale power generation systems. They also offer continuous energy output, in contrast to intermittent sources such as solar and wind, which require storage systems to ensure energy availability. Additionally, ongoing advances in fuel cell materials, cost-reduction strategies, and the global push toward decarbonization have made fuel cells a highly promising alternative within the landscape of renewable energy technologies [89,90,91].

In both energy storage and conversion systems, the cathode compartment typically features a carbon-based electrode, which incorporates a catalyst that must be carefully investigated, developed, and optimized to ensure optimal electrocatalytic performance in the oxygen-reduction reaction (ORR). Numerous studies in the literature have identified the ORR as one of the primary bottlenecks in the advancement of these technologies, largely due to the high cost of catalyst materials, significant overpotentials, and low limiting current densities. To overcome these limitations, platinum (Pt) is the metal noble catalyst involved to ensure the number of electrons necessary to ensure the achievement of ORR, raising issues related to the use of platinum, such as its non-abundance in nature, high cost, and low chemical stability. As a result, the pursuit of novel cathode catalysts has become a prominent scientific and technological focus, as evidenced by the substantial rise in related research publications over the past 15 years [92,93]. The existing studies can generally be divided into two categories: (i) catalysts based on non-precious metals [94,95] and (ii) metal-free catalysts. In the first category, although the use of non-precious metal catalysts has reduced the reliance on noble metals such as platinum, these metals may suffer from leaching and agglomeration, leading to a gradual decline in performance over time [96,97]. Nonetheless, considerable advancements have been made in this area, contributing to the development of highly efficient devices, as documented in several comprehensive reviews [96,98,99,100,101,102,103,104]. The second, and arguably more innovative, approach involves metal-free catalysts derived from heteroatom-doped carbon materials, a specialized subset within the broader study of defects in carbon structures, as summarized in Table 1. The absence of metals in these catalysts offers the potential for substantial cost reductions in their production.

To reduce overall production costs, enable a rapid and flexible fabrication process for graphene-like materials (LIG) suitable for use as electrodes in energy storage and conversion devices, and to allow precise tuning and customization of electrode geometry through defined graphene patterns, direct laser writing (DLW), using CO_2_ laser irradiation, has emerged as a highly promising technique. This method offers a versatile and scalable route for the development of carbon-based electrodes with tailored structural and electrochemical properties. The following sections of this review will provide a more detailed overview of the synthesis of laser-induced graphene (LIG) materials, with a particular focus on their application as sustainable catalysts for the oxygen reduction (ORR) in renewable energy systems.

### 3.2. LIG as Sustainable Catalysts for the Oxygen Reduction Reaction in Renewable Energy Devices

In sustainable and renewable energy devices such as metal–air batteries, fuel cells, and water electrolysis, the oxygen reduction reaction (ORR) results in being one of the main bottlenecks of these technologies, able to limit their industrialization. The ORR mechanism involves the following steps:

(1) $$O_{2}+{4H}^{+}+{4e}^{-}\to {2H}_{2}\;E^{0}=1.229\;V$$

(2) $$O_{2}+{2H}^{+}+{2e}^{-}\to {2H}_{2}O_{2}\;E^{0}=0.695\;V$$

(3) $$H_{2}O_{2}+{2H}^{+}+{2e}^{-}\to {2H}_{2}O\;E^{0}=1.763\;V$$

To overcome the limitations induced by direct-oxygen reduction reaction, noble-metal based catalysts were developed in the last years to overcome the limitations of low kinetic proper of this reduction reactions. In this scenario, Pt-based samples result to be the ideal catalysts towards direct-oxygen reduction reactions, due to their capability to ensure a number of electrons equal to four, avoiding as many intermediate products as possible, like oxygen peroxide (H_2_O_2_), which can be detrimental for the chemical stability of electrodes and for the overall devices’ performance.

During the last ten years, many works in the literature investigated the possibility to substitute for or reduce the amount of platinum required to develop the best catalyst layers for ORR, due to the high cost of platinum and the fact that it is not abundant in the environment [96,97]. In this context, the development of metal-free based catalysts layers, which satisfied some characteristics, such as being highly active, low-cost, and stable electrochemical catalysts, received great interest, aiming to ultimately replace the most active but scarce noble metal catalysts. Among the various alternative catalyst materials explored, carbon-based electrochemical catalysts are considered promising substitutes for noble metal electrocatalysts, as summarized in Table 1. However, their practical application is often limited by either low activity or poor stability. To enhance their ORR catalytic performance, various strategies have been implemented, including the incorporation of foreign elements as dopants and the development of heterostructures. In terms of doping, nitrogen (N) is regarded as a highly desirable heteroatom, as it not only enhances ORR activity but also increases the density of active sites in graphene. Different works in the literature, at the same time, investigated the complicated and contended issues regarding the effective role of heteroatoms to improve the electrochemical properties toward the ORR [96]. The deep analysis conducted by Bermejo et al. allowed concluding that the main advantages of non-doped and doped carbon-based materials, proposed as electrocatalyst layers for ORR, can be identified/found in intrinsic properties, such as porosity and defects. In this context, an important role was covered by the defects, understood as heteroatoms used as active catalytic sites. Notably, the integration of experimental investigations, supported by advanced material characterization, and computational modeling has led to key insights that are now widely recognized within the scientific community. Among nitrogen-doped carbon materials, two nitrogen functionalities are most identified as being responsible for the formation of highly active catalytic sites: pyridinic nitrogen and quaternary (graphitic) nitrogen species. While this is generally accepted, it is essential to emphasize that the precise structural configuration of these nitrogen groups plays a critical role in determining the overall catalytic performance. Variations in local bonding environments, electronic effects, and the degree of integration within the carbon matrix all contribute to differences in electrocatalytic activity. For all these reasons, the implementation of different techniques suitable to tune the presence of heteroatoms inside the carbon-based lattice and engineer the final doped carbon-based materials are vital to improving the electrocatalytic activity of the final layer. In this scenario, the CO_2_ laser writing, implemented for the synthesis of LIG-materials, has been widely discussed in recent years. It is a technology that is in its early stages of development, but at the same time, its rapid development is mainly due to the ability to assist the optimization of the structure within the catalyst and the presence of heteroatoms to improve performance. Indeed, it was important to underline how the CO_2_ laser writing allowed the creation of intrinsic porous-LIG materials. In this context the porosity structures inside the catalyst’s layers affected the formation of triple phase interfaces, which is defined as the place on which the electrons, ions, and oxygen molecules recombined one with each other, leading to direct oxygen reduction reaction. The higher the triple phase interface, the higher the overall electrocatalytic performance of catalyst layers. As deeply explained in the previous paragraph, at the same time, many strategies can be employed to induce the formation of heteroatoms inside LIG materials, making them a good candidate for a metal-free carbon-based electrode for energy storage and conversion devices.

### 3.3. LIG for Proton Exchange Membrane Fuel Cells (PEMFCs)

**Figure 13:**
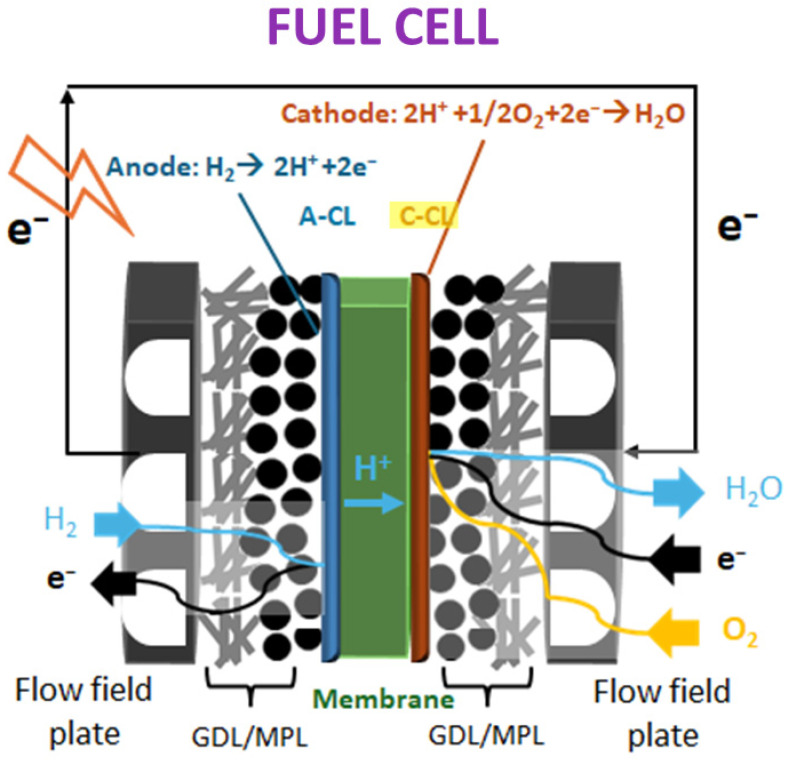
Scheme of main components of fuel cells and electrochemical reactions involved.

PEM (polymer electrolyte membrane) fuel cells have received great attention from the research and industrial worlds due to their capability of reducing energy use, pollutant emissions, and dependence on fossil fuels [121,122,123,124,125]. PEM fuel cells, whose main components are represented in Figure 13, are designed by involving a polymer electrolyte membrane, commonly Nafion, as a proton conductor and electrochemical catalysts for electrochemical reactions, occurring at low temperature.

LIG-based cathodes offer significant advantages in water management for PEM fuel cells, a critical factor in their efficiency and longevity. The hierarchical porosity of LIG facilitates improved oxygen diffusion and water removal, preventing flooding and ensuring consistent performance. Additionally, functionalized LIG structures can optimize the hydrophilic/hydrophobic balance within the catalyst layer, further enhancing water management. Future studies should focus on integrating LIG with advanced membrane materials and exploring the long-term stability of LIG-based cathodes under real-world operating conditions.

Optimizing water management in LIG-based PEMFC cathodes remains a significant challenge. While the porous structure of LIG offers natural advantages in gas diffusion, its interaction with water at different operational conditions requires further investigation. Future research should address the design of multi-layered LIG architectures that enhance water evacuation while maintaining optimal proton conductivity. Additionally, long-term cycling tests are needed to evaluate the durability of LIG-based cathodes in high-humidity environments. During the last ten years, many efforts were focused on the investigation and development of microfluidic fuel cells. In this scenario, Rao et al. [121] proposed a cost-effective microfluidic fuel cell with LIG electrodes. Various parameters, including fluid concentration, catalyst type, and flow rate, were analyzed to optimize fuel cell performance. The LIG electrodes were fabricated on a polyimide substrate using CO_2_ laser irradiation and integrated into a polydimethylsiloxane (PDMS) microchannel, produced via conventional soft lithography. In this setup, the LIG electrodes functioned as both the anode and cathode, with atmospheric oxygen serving as the oxidant, while formic acid and sulfuric acid acted as the fuel and electrolyte, respectively, to facilitate ionic exchange under co-laminar flow. To further enhance performance, the LIG electrodes were modified with different catalyst materials, such as Ag nano ink, SWCNT, MWCNT, MnO_2_, and TiO_2_. The optimized fuel cell, utilizing Ag nano ink as a catalyst, achieved a maximum open-circuit potential of 740 mV, a peak current of 765.06 μA/cm^2^, and a power density of 88.80 μW/cm^2^ at a flow rate of 24 mL/h.

### 3.4. LIG for Microbial Fuel Cells and Enzymatic Biofuel Cells

In microbial fuel cells (MFCs) and bioenzyme fuel cells (EBFCs), the availability of cost-effective cathode catalysts to support an ORR reaction is of paramount importance. In recent years, considerable efforts have been made to improve the catalytic performance while keeping the cost low and the fabrication process easily scalable. In this direction, LIG-type graphene represents an excellent catalytic option with excellent physicochemical properties while offering a viable and simple technological alternative to the conventional, complex, and expensive graphene fabrication methods.

Senthilkumar et al. investigated the performance of LIG obtained from polyether sulfone (PES)-modified carbon (CC) fabric to develop a cathode catalyst useful in wastewater treatment in MFC-type systems.

Using cyclic voltammetry and electrochemical impedance spectroscopy, they evaluated the charge transfer capacity and electrocatalytic activity of the so-synthesized material. The material, named PES-LIG-CC, was used as an electrode in MFC, providing a potential two times higher than that of the pure CC catalyst, although lower than that of Pt/C, but with a maximum chemical oxygen demand removal of 84% after three cycles, demonstrating the high potential of LIG for this application context [122].

The development of cost-effective and scalable bioelectrodes with optimal physical properties remains a key challenge in achieving automated and reliable enzymatic biofuel cells (EBFCs). Rewatkar et al. [123] investigated the fabrication of customized CO_2_ laser-induced flexible graphene (LIFG) bioelectrodes on a polyamide substrate. The affordability and adaptability of LIFG bioelectrodes were further demonstrated for EBFC applications by integrating them into a microfluidic device, fabricated using conventional soft lithography on polydimethylsiloxane (PDMS). Initially, LIFG bioelectrodes were produced using optimized CO_2_ laser parameters (power and speed), followed by material characterization to confirm the presence of graphene. The surface morphology of the untreated polyamide sheet, LIFG, and enzyme-modified LIFG bioelectrodes (functionalized with GOx and laccase) was then analyzed. Comprehensive voltametric electrochemical studies were conducted on the modified LIFG bioelectrodes before integrating them into the microfluidic device. The system achieved a power density of 13 μW/cm^2^ (52 μA/cm^2^) at an optimized flow rate of 200 μL/min. This fabrication approach demonstrates the strong potential of LIFG bioelectrodes for electrochemical redox reactions and polarization performance within a microfluidic environment. Furthermore, power output could be significantly enhanced by incorporating additional cofactor-based electrochemistry and device stacking strategies.

Jayapiriya and coworkers [124] fabricated a one-step LIG bioelectrodes for EBFC application, successfully integrating them into a PDMS microfluidic device. The overall manufacturing process was simple and quick, eliminating the need for any further post-processing. LIG electrodes were created with the addition of multi-walled carbon nanotubes (CNT), in order to fabricate C-LIG electrodes, which offered improved performance and enzyme stability. CNT-functionalized LIG electrodes were integrated into microfluidic fuel cells to ensure laminar flow. The microfluidic device housing the novel C-LIG bioelectrodes, without added catalysts, generated 2.2 μW/cm^2^ at a flow rate of 200 μL/min, increasing the performance of the LIG bioelectrodes by 1.37-fold. The authors predicted further improvement in power output by optimizing the nano-functionalization of the LIG [124].

### 3.5. LIG Materials as Positive Cathode for Energy Storage Systems

#### Metal–Air Batteries

One of the key components in the design of rechargeable zinc–air batteries is the cathode catalyst, which is used to facilitate oxygen reduction and evolution reactions (ORR and OER). Ren et al. [125] reported the synthesis of hybrid metal oxide/graphene catalysts in a flexible graphene film formed in situ by laser induction. The authors combined ORR-active Co/Mn catalysts with OER-active Ni and Fe species to promote bifunctional activity toward both reactions. The resulting batteries obtained from these LIG-based catalysts exhibited a high peak discharge power density of 98.9 mW cm^−2^, an energy density of 842 Wh/kg_Zn_, and a high reversibility and durability with charge/discharge cycles exceeding 200 h. These hybrid catalysts outperform precious metal-based catalysts such as Pt and RuO_2_ in zinc–air batteries, opening the possibility of exciting applications for wearable and flexible electronic devices [125].

The same author also presented the synthesis of highly efficient bifunctional OER/ORR catalysts based on laser-induced direct graphene (LIG) process to produce Co_3_O_4_/LIG systems, where the LIG material was decorated with Co_3_O_4_. The Co_3_O_4_/LIG showed promising performance in Zn–air and Li-O_2_ batteries. In particular, the rechargeable Zn–air battery provided an open-circuit potential of 1.46 V and a power density of 84.2 mW/cm^2^ at 100 mA/cm^2^, while the Li-O_2_ battery with Co_3_O_4_/LIG cathode exhibited excellent stability of up to 242 cycles and low overpotentials. The novel Co_3_O_4_/LIG electrode showed OER and ORR activity comparable to that of noble metal-based catalysts [126].

Similarly, a lithium-ion battery was successfully fabricated by decorating LIG with MnNiFe to obtain highly efficient bifunctional OER/ORR catalysts. The resulting MnNiFe/LIG electrodes showed promising performance in Li-O_2_ and Li–air batteries without a redox mediator. The resulting Li-O_2_ battery with MnNiFe/LIG catalysts was discharged/charged for 150 cycles with a slight increase in discharge potential of about 0.24 V. The breathable Li–air battery with MnNiFe/LIG catalyst maintained its stability up to 350 cycles [127,128].

Ren and coworkers investigated a novel strategy to increase stability of Li-O_2_ batteries, reaching cycling capacity up to 2 mAh/cm^2^. The authors proposed to combine a double polymer gel electrolyte (DPGE) as a quasi-solid electrolyte, specifically designed to reduce probability of possible side reaction with superoxide intermediates, with a highly effective Mn-based catalyst, prepared by direct laser writing on polymers. The novel quasi-solid Li-O_2_ batteries fabricated by this facile and fast processing route, were highly reversible, without short circuits or increased interfacial resistance for over 2000 h and with stable galvanostatic charge/discharge performance for over 200 cycles (2000 h) with a cut-off capacity of 0.4 mAh/cm^2^ [129].

In their work, Aldhafeeri et al. [130] demonstrated a simple approach to reduce the Pt content to less than 2 wt% for ORR catalysis by interfacing Pt with CoOx nanoparticles arranged within a highly conductive laser-induced-graphene (LIG) matrix. Polymerized furfuryl alcohol, preloaded with Co and Pt precursors, was laser-treated to induce carbonization of the material. The process resulted in a mixture of spherical PtCoOx nanoalloys, consisting of core (CoOx) and shell (Pt) structures. This LIG-PtCoOx electrode closely matched a Pt/C reference, exhibiting low onset and half-wave potentials in an alkaline environment. The effectiveness of LIG-PtCoOx was demonstrated by testing the material as an air cathode for zinc (Zn)–air batteries, achieving improved stability (118 h of operation) and rechargeability (0.75 V voltage gap), with higher peak power density compared to commercial reference Pt/C cathode-based batteries.

## 4. Conclusions

This review presents the implementation of new technological process, suitable to synthesize, design, and optimize laser-induced graphene materials as catalyst layers for ORR. It was important to highlight several key inconsistencies in the literature, such as variations in electrical conductivity and surface area, which often stem from differences in laser parameters (e.g., wavelength, pulse duration, power density) and precursor materials (e.g., polyimide, lignin-based substrates). These discrepancies point to a lack of standardized synthesis protocols, complicating direct comparisons across studies. At the same time, this review has emphasized unresolved questions regarding the mechanisms underlying LIG formation, particularly the competing roles of photothermal vs. photochemical processes and the extent to which they influence porosity, graphitization degree, and defect density. There are also ongoing debates in the field regarding the contribution of edge defects and heteroatom doping (intentional or substrate-derived) to the observed electrochemical and sensing performance of LIG. The CO_2_ laser writing, implemented for the synthesis of LIG-materials, has been widely discussed in recent years. It is a technology that is in its early stages of development, but at the same time its rapid development is mainly due to the ability to assist the optimization of the structure within the catalyst and the presence of heteroatoms to improve performance. Indeed, it was important to underline how the CO_2_ laser writing allowed the creation of intrinsic porous-LIG materials. In this context the porosity structures inside the catalyst’s layers affected the formation of triple phase interfaces, which is defined as the place on which the electrons, ions, and oxygen molecules recombined one with each other, leading to direct oxygen reduction reaction. The higher the triple phase interface, the higher the overall electrocatalytic performance of catalyst layers. As explained in the previous paragraph, at the same time, many strategies can be employed to induce the formation of heteroatoms inside LIG materials, making them a good candidate for metal-free carbon-based electrodes for energy storage and conversion devices. LIG represents a promising class of metal-free electrocatalysts for ORR due to its unique physicochemical properties, tunability, and cost-effectiveness. Heteroatom doping and surface functionalization have demonstrated significant improvements in ORR performance, bringing LIG closer to practical applications in fuel cells and other electrochemical energy systems. However, challenges related to scalability, stability, and performance consistency must be overcome to achieve commercial viability. Future research should focus on optimizing LIG synthesis, exploring novel doping strategies, and integrating LIG-based catalysts into functional energy conversion devices.

Unlike conventional graphene production methods, LIG does not require complex chemical processing, making it a more environmentally friendly and scalable alternative. Furthermore, its compatibility with flexible and lightweight substrates enables the development of wearable and portable energy devices.

Recent advances in LIG research have expanded its potential applications beyond traditional energy storage. Researchers have successfully integrated LIG into hybrid materials, improving its mechanical stability and electrochemical performance. Moreover, modifications such as heteroatom doping and surface functionalization have been explored to tailor its properties for specific applications, including electrocatalysis and sensor technologies.

Despite its many advantages, several challenges remain in the widespread adoption of LIG-based materials in commercial energy devices. Issues such as mechanical integrity under long-term operation, electrochemical stability in harsh environments, and mass production scalability need to be addressed.

This review provides an overview of recent advances in LIG synthesis methods, its physicochemical properties, and its integration into various electrochemical energy storage and conversion devices. Furthermore, we discuss the challenges and future opportunities for optimizing LIG-based materials in next-generation energy applications.

## Figures and Tables

**Table 1:** All possible Doped-carbon based electrocatalysts, obtained by starting different precursors.

Carbon-Based Materials as Electrochemical Active Catalyst Layers for ORR					
	Typer of Catalyst Layers	Onset Potential	Number of Electrons	Electrolyte Solution	Reference
Doped carbon electrocatalysts derived from organic waste	Efficient ORR catalysts obtained by pyrolysis implemented on waste-tire	−90 mV	4 e^−^	Alkaline solution for fuel cells and metal air batteries	[105]
	Tire waste as source for the synthesis of heteroatoms doped carbon nanosheets (CNS)	−0.217 V to −0.220 V	–	–	[106]
	Sewage sludge rich in N and P	−0.04 V	4 e^−^	Alkaline solution	[107]
	Fe-modified mesoporous N-doped carbons	0.890 V	–	–	[108]
N-doped carbon based catalysts derived from soybeans	Honeycomb-like Fe–N co-doped porous carbon material	0.886 V in acidic conditions; 0.989 V in alkaline solutions	–	Both alkaline and acidic solutions	[109]
Doped and/or Co-doped carbon based electrocatalysts	P-doped porous carbon	0.96 V	–	–	[110]
	N and F co-doped carbon nanofibers	0.94 V	4 e^−^	–	[111]
	Triazine polymers as self-doping N, F, and P carbon based catalysts	0.93 V	–	Both alkaline and acidic solutions	[112]
	Sulphur and nitrogen co-doped graphene	from −0.11 V to −0.13 V	–	Alkaline solution	[113]
	N-doped porous graphene with a certain amount of carbon, obtained starting from pyrolysis implemented on glucose	0.91 V vs. RHE	Close to 4 e^−^		[114]
	N-doped graphene obtained by implementing a thermal annealing treatment obtained from graphene oxide mixed with melamine	−0.1 V vs. RHE	Equal to 3.4 e^−^–3.6 e^−^	Alkaline solution: 0.1 M KOH	[115]
	Phosphorous-doped graphite	0.1 V vs. RHE	Close to 3 e^−^	Alkaline solution: 0.1 M KOH	[116]
	Co-doping of P and N carbon materials	0.94 V vs. RHE	Close to 4 e^−^	Alkaline solution: 0.1 M KOH	[117]
	Co-doping of Born and Nitrogen graphene N-co-doped carbon nanotubes	The onset potential achieved is similar to the one obtained by Pt/C catalyst layer	Equal to 3.97 e^−^	Alkaline solution: 0.1 M KOH	[118]
	Sulfur (S)-doped graphene	close to −0.15 V vs. RHE	equal to 3.81 e^−^	Alkaline solution: 0.1 M KOH	[119]
	Co-doped P-N sites in carbon nanotube and graphene foam	0.91 V close to 20 wt% of Pt	close to 4 e^−^	Acidic media	[120]
